# Data to Decisions: Methods to Create Neighbourhood Built Environment Indicators Relevant for Early Childhood Development

**DOI:** 10.3390/ijerph19095549

**Published:** 2022-05-03

**Authors:** Karen Villanueva, Amanda Alderton, Carl Higgs, Hannah Badland, Sharon Goldfeld

**Affiliations:** 1 Centre for Urban Research, Royal Melbourne Institute of Technology (RMIT) University, Melbourne, VIC 3000, Australia; amanda.alderton@rmit.edu.au (A.A.); carl.higgs@rmit.edu.au (C.H.); hannah.badland@rmit.edu.au (H.B.); 2 Murdoch Children’s Research Institute, Parkville, VIC 3052, Australia; sharon.goldfeld@rch.org.au; 3 Department of Paediatrics, The University of Melbourne, Parkville, VIC 3052, Australia

**Keywords:** built environment, data linkage, early childhood development, neighbourhood, indicators

## Abstract

Healthy development in the early years lays the foundations for children’s ongoing physical, emotional, and social development. Children develop in multiple contexts, including their local neighbourhood. Neighbourhood-built environment characteristics, such as housing, walkability, traffic exposure, availability of services, facilities, and parks, are associated with a range of health and wellbeing outcomes across the life course, but evidence with early years’ outcomes is still emerging. Data linkage techniques were used to assemble a dataset of spatial (objectively-measured) neighbourhood-built environment (BE) measures linked to participant addresses in the 2015 Australian Early Development Census (AEDC) for children living in the 21 most populous urban and regional Australian cities (n = 235,655) to help address this gap. This paper describes the methods used to develop this dataset. This linked dataset (AEDC-BE) is the first of its kind worldwide, enabling opportunities for identifying which features of the built environment are associated with ECD across Australia at scale, allow comparisons between diverse contexts, and the identification of where best to intervene. National data coverage provides statistical power to model real-world complexities, such as differences by city, state/territory, and remoteness. The neighbourhood-built environment can be modified by policy and practice at scale, and has been identified as a way to help reduce inequitable early childhood development outcomes.

## 1. Introduction

Major global agencies, including the World Health Organization and UNICEF, recognise early childhood (0–8 years) as one of life’s critical development periods [1] that lays the foundations and sets the trajectories for children’s present and future wellbeing. [2]. Healthy development includes salient, but interrelated, aspects, such as physical development, social competence, emotional maturity, language and cognitive development (e.g., school-based academic learning), and general knowledge and communication [3]. The early years is a time when one’s environment can critically influence how the brain develops [4]. Children with stimulating and positive environments early in life have optimal foundations for their ongoing physical, social, emotional, and cognitive development [5]. For example, they develop skills in learning, communicating, problem-solving, and decision-making [6,7].

Setting optimal child development trajectories includes understanding the multiple factors shaping healthy development, including children’s individual characteristics, family, and the environments (both social and physical) in which they are raised [8].

The majority of child development research has focused on the influence of family and school environments, and has largely ignored the neighbourhood [9]. The neighbourhood environment is recognised as an important social determinant of early childhood outcomes, including the daily living conditions in which children grow and develop [10,11]. Neighborhoods have important exposures and resources that impact child development, with rich sources of stimulation and opportunities to explore, learn, and interact with others and their surroundings [7,12]. The neighbourhood setting, comprising its design and built environment, includes features such as housing type and layout, street design, traffic, parks, child care facilities, and other infrastructure and services [9]. Neighbourhood-built environment attributes can be modified by policy and practice, meaning that finding the right leverage points can likely have relatively large, wide-ranging, and on-going effects, particularly when targeting whole-of-population early childhood development (ECD) outcomes [9].

Mounting evidence shows associations between neighbourhood features and older children’s behaviours (e.g., physical activity [13] and sitting time [14]) and health (e.g., obesity) [15,16,17], but the relationship between the built environment and ECD is largely unexplored [18,19,20]. This is despite socioecological frameworks of ECD [21,22] and previous research suggesting features of the local environment in which families live have an important influence on parents′ capacity to raise their children and, therefore, promote good developmental outcomes [23,24].

Although research on the built environment and ECD is nascent, the strongest evidence to date exists for associations between geographic disadvantage and ECD outcomes. Disadvantaged neighbourhoods are most vulnerable to poorer early childhood outcomes [25]. For example, differences in area-level disadvantage have translated into inequities in ECD outcomes such as developmental delay in language [26], and behavioural and mental health problems [27]. Alongside this research, place-based and health studies suggest the distribution and quality of built environment features differ between neighbourhoods; more disadvantaged areas generally have poorer access to quality services and destinations compared with more advantaged areas [28].

In the last few years, research reviews and viewpoints highlight the need to target neighbourhood effects research on ECD [19,20,25,29]. The substantial international interest in relationships between early childhood, urban design and planning, and place-based strategies is also reflected in national and global policy agendas on ECD, liveability, and child-friendly cities initiatives [30,31,32], and emphasised in the Lancet Commission on Child Health and Wellbeing paper on the investment in children’s futures [18]. Moreover, the UN Sustainable Development Goals (SDGs) call for equitable access to quality early education (Goal 4), reduced inequalities (Goal 10), and sustainable cities and communities (Goal 11) [33]. Though all these initiatives advocate the neighbourhood environment as a mechanism to promote ECD and wellbeing, and reduce inequities, evidence-based metrics and data such as indicators are needed to monitor progress towards this ambition. Internationally, there are no theoretically- or empirically-derived spatial indicators to monitor and guide urban planning policies and/or interventions that support good ECD outcomes and reduce inequitable conditions in the first place. Australian research with adults [34] shows that evidence-based metrics such as indicators are valuable policy and planning tools to benchmark and monitor neighbourhood progress. For example, the National Cities Performance Framework Dashboard [35] has adopted indicators such as ‘access to services’ (e.g., health infrastructure access index), and ‘getting to work’ (e.g., proportion of journeys to work by public transport); these indicators aim to assist all levels of government, industry, and the community to better target, monitor, and evaluate policy and investments. Though indicators in the ECD context are currently used to monitor factors such as infant mortality [36], school enrolment [37], and immunisations [38,39], there are limited targeted decision support analytics and tools (e.g., indicators) that can drive or inform designing ‘child-friendly’ neighbourhoods at scale.

We know from other health- and place-based research that spatial measures of the neighbourhood-built environment (e.g., access to green space) have been linked to population datasets to explore objective relationships with well-being across the life course [40]—in children [41], adolescents [42], adults [34], and older adults [43]. Methodological capabilities (e.g., data linkage, Geographic Information Systems (GIS) software, SoftGIS [44]), and big data population datasets have only recently become available to pursue this, particularly in the ECD space [45]; the opportunities these methods afford have important implications for ECD research. For example, linking spatial (objective) built environment data to ECD data allows the opportunity to explore objective built environment relationships with ECD outcomes, a key gap in neighbourhood effects on ECD research. This methods paper aims to describe the data and linkage methods used to develop a national ECD dataset linked to numerous conceptually-informed, spatially-attributable neighbourhood-built environment indicators calculated for unique neighbourhoods for every child living in the 21 largest Australian cities (the Australian Early Development Census (AEDC)—Built Environment (BE) dataset, i.e., AEDC-BE). A secondary aim is to articulate the challenges and strengths of the AEDC-BE dataset.

## 2. Materials and Methods

Spatial neighbourhood-built environment measures were linked to unique home addresses of children from the 2015 Australian Early Development Census (AEDC) residing in Australia’s largest (most populous) 21 cities. Australia’s largest 21 cities consist of eight capital cities (Adelaide, Brisbane, Canberra, Darwin, Hobart, Melbourne, Perth, Sydney) and 13 major cities with a population of ≥80,000 (which includes major regional cities—Albury—Wodonga, Ballarat, Bendigo, Cairns, Geelong, Gold Coast-Tweed Heads, Launceston, Mackay, Newcastle-Maitland, Sunshine Coast, Toowoomba, Townsville, Wollongong), plus Western Sydney [35]. Smaller regional (≤80,000 people) and remote areas were excluded, as our built environment measures are typically conceptualised and applied to more urbanised areas. The cities span across all of Australia’s states and territories: Queensland, New South Wales, the Australian Capital Territory, Victoria, South Australia, Western Australia, Northern Territory, and Tasmania. Australia is the only country internationally to have an ECD ‘census’, meaning it has an overall participation rate of 96.5% of children at school entry [3], and makes the AEDC Built Environment (AEDC-BE) linked dataset a globally unique dataset. The AEDC-BE linked dataset is cross-sectional, meaning that it offers a ‘snapshot’ of child development (taken in 2015) and children’s neighbourhood-built environments. First, we describe the data linkage process. Second, we describe the Australian Early Development Census (ECD data and measures). Third, we describe the built environment data and measures.

### 2.1. Data Linkage Process

**Figure 1:**
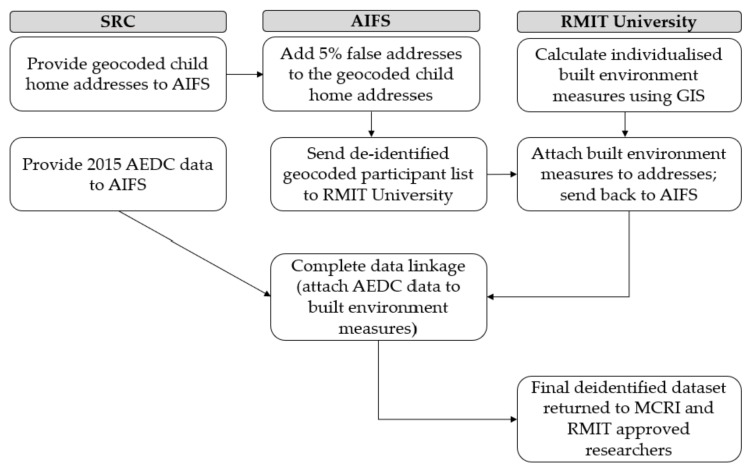
Steps in the AEDC-BE data linkage process. AEDC: Australian Early Development Census. AIFS: Australian Institute of Family Studies. MCRI: Murdoch Children’s Research Institute. SRC: Social Research Centre.

The data linkage process (Figure 1) ensured data separation principles between the data custodians, data linkage agency, and research teams. The Social Research Centre (SRC) through the Australian Government Department of Education Skills and Employment (DESE (data custodians), provided the 2015 AEDC data and geocoded addresses (latitude/longitude coordinates) of 2015 AEDC participants were to the Australian Institute of Family Studies (AIFS), an approved data linkage body. AIFS provided a de-identified AEDC participant list of geocoded addresses that included an additional 5% false addresses to RMIT University, to help ensure anonymity. The built environment spatial measures (e.g., distance from home to the closest park) were calculated around each geocoded point (i.e., home address), attached to the dataset of geocoded addresses, and returned to AIFS. AIFS then removed the false addresses, dropped cases that did not fall within the 21 Australian cities, de-identified the final linked dataset by removing the geocodes, and integrated the spatial built environment measures with AEDC content data (e.g., child development outcomes). The final de-identified linked dataset was then provided to the research team for analysis (August 2019).

Approvals were obtained from the Royal Children’s Hospital (RCH) Human Research Ethics Committee (HREC) (#30016), with registration at RMIT University HREC (#20749), AEDC data custodians (180130C), and the authorised data linkage agency (AIFS). A memorandum of understanding between AIFS and DESE was also undertaken.

### 2.2. Early Childhood Development Data and Measures

The AEDC is an internationally validated and reliable Australian child population measure of ECD, adapted from the Canadian Early Development Index [46]. The AEDC provides teacher-reported national data on five salient and interrelated child development domains: physical health and wellbeing, social competence, emotional maturity, language and cognitive skills (school-based), and communication skills and general knowledge [3]:

(1) Physical health and wellbeing refers to children’s physical readiness for the school day, physical independence, and fine motor skills.
(2) Social competence refers to children’s overall social competence, responsibility and respect, approach to learning, and readiness to explore new things.
(3) Emotional maturity refers to children’s pro-social and helping behaviours, and absence of anxious and fearful behaviour, aggressive behaviour, and hyperactivity and inattention.
(4) Language and cognitive skills (school-based) refers to children’s basic literacy, and interest in literacy, numeracy and memory, advanced literacy, and basic numeracy.
(5) Communication skills and general knowledge refers to children’s communication skills and general knowledge based on broad developmental competencies and skills.

Children were scored between 0 and 10 on each of the five developmental domains; higher scores indicate better developmental status [3]. Each domain was subsequently categorised as: ‘developmentally vulnerable’ (≤10th centile), ‘developmentally at risk’ (11th to 25th centile), or ‘developmentally on track’ (≥26th centile) based on 2009 cut-off scores. The cut-off scores established in 2009 provide a reference point against which later AEDC results can be compared, and they have remained the same across all collection cycles [3]. Children who were developmentally vulnerable on one or more of the AEDC domains (DV1) are typically publicly reported, and thus used in this paper.

The AEDC triennial data collections are funded every three years by the Australian Government, with the first national roll-out occurring in 2009. The 2015 AEDC data were obtained for this research; it was the first of the data collections to have maternal education included in the survey. Maternal education is a commonly used key marker of children’s/families’ socioeconomic circumstances [47], and has been associated with children’s developmental outcomes and wellbeing [48]. The AEDC records information about the child’s age, sex, special needs status, indigenous status, English as a second language, and other variables [3], which can be accounted for in any analyses. Neighbourhood socioeconomic context (i.e., whether a child resides in a disadvantaged neighbourhood) and remoteness are also included in the AEDC. Neighbourhood disadvantage was measured using the 2016 Socioeconomic Index for Areas—Index of Relative Socioeconomic Disadvantage (SEIFA-IRSD), which is a composite variable comprising of 16 items, and created by the ABS using Australian Census data [49]. This information is available in the dataset at SA1-level [50]. Remoteness of the child’s residence was classified into five categories (major city, inner regional, outer regional, remote, or very remote) according to the Australian Statistical Geography Standard Remoteness Structure [50]. Information about the AEDC is available: https://www.aedc.gov.au (accessed on 8 March 2022).

### 2.3. Built Environment Data and Measures

Conceptualising and creating over 80 ECD-relevant built environment measures (see Table 1 for examples) was informed by a ‘child liveability’ work program, drawing upon earlier reviews [9,19] and the Kids in Communities Study (KiCS) [51]. Through KiCS, numerous ‘Foundational Community Factors’ that plausibly lay the foundations of an optimal community for young children were identified. Parks (a type of public open space), public transport, traffic safety, walkability, facilities and services, and housing emerged as important built environment factors or ‘domains’, and were primarily informed by qualitative findings from 25 communities across five Australian states and territories [52]. One key recommendation of KiCS was to develop better quantitative indicators for ECD; this was supported by the federal and state government partner organisations on the project. The development of the linked AEDC-BE dataset of quantitative built environment objective measures to early childhood outcomes is an important first step toward achieving this.

The built environment domains in Table 1 thus reflect the KiCS foundational community factors related to the built environment. Each built environment domain included a range of different spatial measures, such as count (number) of, and distance (in metres) to, different types of facilities and services, such as libraries and maternal child health centres. For some services, quality measures were also included, e.g., distance to closest early childhood education, and care centre meeting Australian standards. Park features were included (e.g., presence of toilet, playground) to derive park ‘quality’ measures. Further information is available in the metadata section of the Australian Urban Observatory [53] (https://auo.org.au/portal/metadata) (accessed on 8 March 2022).

Spatial measures were created at the parcel level (i.e., child′s residential address); this is the smallest geography possible. In the absence of obtaining accurate accounts of where children visit (e.g., global positioning system technology), ‘best practice’ representation of children’s ‘neighbourhoods’ are typically by network buffers (along the street or pedestrian network) ranging from 400 m to 1600 m (approximately 5–20 min walking time) around their home, provided home addresses can be obtained and geocoded. To account for the likely presence of some destinations at larger area scales (e.g., less common that children have ECEC services [55] and family-friendly destinations, such as public libraries and community centres within 1600 m of home), 3200 m was used for some features. Children participating in the AEDC were ‘matched’ to the dwelling sample point nearest their home; this sample point was used to calculate individualised built environment measures for each child. Address points from the Geocoded National Address File (G-NAF) [56] located within study region boundaries for the 21 cities were used as dwelling sample points located in a Mesh Block (smallest geographical area defined by the ABS ASGS; range, 30–60 dwellings/area) with a positive 2016 dwelling count [57]. Dwelling sample points within each study region were considered valid proxies for linkage if the following criteria were satisfied: in an SA1 (Statistical Area Level 1; average of 400 persons/area and the smallest geographic boundary provided by the Australian Bureau of Statistics) [50], with recorded 2016 SEIFA Index of Relative Socio-Economic Disadvantage score [49], and located within 500 m distance of an AEDC participant.

Workflows to create the built environment measures were scripted using Python, with network analyses for distances to destinations conducted using ArcGIS network analysis. Additional spatial analyses and overall data management were undertaken using a PostgreSQL and PostGIS.

Data sources (Supplemental Material, Table S1) for the spatial measures in Table 1 were collated largely from open-source data, and national data were used to ensure consistency across Australia. Data sources included: Australian Bureau of Statistics (ABS) Australian Census (2016); OpenStreetMap; the Australian Children’s Education & Care Quality Authority (ACECQA); and the National Health and Services Directory (NHSD). OpenStreetMap is a community-contributed global database of geographic information available to use under an open license. It was used as a source for national road data, open space, and for destinations where alternative nationally consistent sources were not available [58]. Road networks and destinations data were sourced and retained where located within a 10 km Euclidean distance buffer of study region boundaries. The National Quality Standard (NQS) assesses Australian ECEC and outside-school-hours care services against seven quality areas that are important outcomes for children, including educational program and practice, children’s health and safety, physical environment, staffing arrangements, relationships with children, and collaborative partnerships with families and communities [59]. These services are assessed against each of the seven quality areas in the NQS and given an overall rating based on these results (e.g., meeting NQS, exceeding NQS).

Though the primary aim of this paper is to describe the data and data linkage methods used to link spatial built environment measures to ECD data, we have included some descriptive statistics in the results section to illustrate an example of the sample characteristics. Descriptive statistics for the sample were computed using Stata v16. Major (n = 182,913), and regional city samples (n = 22,117) were shown separately for the built environment characteristics.

## 3. Results

The full 2015 AEDC cohort consists of 302,003 children: 96.5% of the estimated five-year old Australian child population. Of the 319,503 addresses provided, 248,744 of supplied address points (including dummy addresses to preserve anonymity) were linked with address-level spatial-built environment measures, with a median match distance of less than 2 m (99th percentile of 64 m; range of 0 to 499 m). As the AEDC-BE dataset does not include children living rural or remote areas, the final linked dataset contains records for 235,655 children living in the 21 largest cities, 78% of the 2015 AEDC cohort [60]. These children are nested within 12,541 classrooms, 4646 schools, 40,046 SA1 ‘neighbourhoods’ [50], 1521 ‘suburbs’ (Statistical Area Level 2, approximately 10,000 persons/area on average) [50], 171 local government areas (municipalities), 21 cities, and 8 states and territories.

Demographic characteristics of the AEDC-BE cohort are summarised by developmental vulnerability status (DV1) and overall in Table 2. Overall, 47,416 children—about 1 in 5 children—were assessed by their teachers as being developmentally vulnerable on at least one domain of child development. Approximately 29% of those who were developmentally vulnerable on at least one domain lived in the most disadvantaged areas, compared to 14.8% living in the least disadvantaged areas. A higher proportion of Aboriginal and Torres Strait Islander children, children who have a language background other than English, or children whose parent’s highest level of education was Year 9 or less were classified as DV1.

In the Kids in Communities Study, one of the Foundational Community Factors related to the physical (built) environment included ‘Facilities and Destinations—Availability and Diversity′; qualitative interviews and focus groups with communities suggested having a range of family-friendly destinations and activities is important for families with young children. With the AEDC-BE dataset, we were able to explore the proportion of children with no access within 1600 m and 3200 m distances to destinations and services locally in major and regional cities by neighbourhood disadvantage.

A higher proportion of children living in regional cities (compared to major cities) had no access to ECEC services exceeding national standards within 3200 m (33.7% regional vs. 5.0% major city), public open space (12.7% regional vs. <1% major city), or a playground (45.9% regional vs. 10.0% major city) within 1600 m. Both major and regional cities have high proportions of children with no public swimming pools (56.1% regional vs. 34.8% major city), public libraries (62.6% regional vs. 33.1% major city), and community centres (73.8% regional vs. 48.9% major city) available within 3200 m, necessitating travelling further to access family-oriented activities.

The distribution of children with no local access to the above destinations and services did not show a social gradient in the direction expected; a higher proportion of children in the least disadvantaged areas had no local destinations. By way of example, in major cities, though the vast majority of children from all types of neighbourhoods had some access to high-quality ECEC services (only 5% had no access to high-quality ECEC), there was an inverse association with neighbourhood disadvantage in terms of no access. That is, compared with the most disadvantaged, a higher proportion of children living in the least disadvantaged areas had no access to high-quality ECEC in their neighbourhood (2.5% of children in the most disadvantaged quintile vs. 4.9% in the least disadvantaged quintile). For regional cities, 14.8% vs. 41.6% of children had no ECEC services exceeding national standards for the most disadvantaged and least disadvantaged neighbourhoods, respectively.

## 4. Discussion

Understanding what, how, how much, and where the neighbourhood-built environment influences child health and development is needed to build evidence to inform urban design interventions to (re)design the neighbourhoods in a way that supports the health needs of young children and their families. Although data linkage methods are not new, progressing this interdisciplinary research field connecting early childhood with urban design and planning is now possible with the increasing availability and use of spatial software and data linkage techniques to link neighbourhood measures with child health data [45]. Key challenges of data linkage methods of built environment to ECD data include: defining the ‘neighbourhood’ unit, maintaining data security and confidentiality during data linkage, the use of non-context and behaviour-specific data, and disentangling cause and effect.

### 4.1. Strengths and Potential of AEDC-BE to Address Key Gaps in Neighbourhood Effects and ECD Research

The AEDC-BE can add to the international neighbourhood effects literature on ECD in three ways. First, interrogating the AEDC-BE dataset will add to the evidence base on built environment effects on ECD by examining built environment features that are associated with ECD inequities.

It is well known that children living in more disadvantaged neighbourhoods typically have poorer ECD outcomes [25]; this is consistent with international social gradient evidence [61], and is also reflected in our Table 2 findings. For example, in 2015, Australian children living in the most disadvantaged areas were over four times more likely to be developmentally vulnerable on at least one of the five Australian AEDC domains relative to children living in the least disadvantaged areas [62]. Goldfeld and colleagues (2018) examined the association between exposure to four lenses of disadvantage (sociodemographic, geographic environments, health conditions and risk factors, and a composite of these) from 0–9 years and child development at 10–11 years using data from the Longitudinal Study of Australian Children. They found children in the most disadvantaged composite trajectory had seven times higher risk of poor outcomes on two or more developmental domains compared with those most advantaged [63]. Exposure to the most advantaged trajectory across all lenses could reduce poor developmental outcomes by as much as 70%. This suggests the need to account for, where possible, measures of different types or ‘lenses’ of disadvantage, including geographic disadvantage [64].

Emerging research shows that neighbourhood- or community-level factors, such as having local resources and amenities, can influence early childhood outcomes [52,65]. For example, previous research shows that successful ECD outcomes partly depend on the availability and quality of ECEC programs [66]. Recent studies using Western Australian AEDC data also found small associations between different types of built environment measures and specific AEDC sub-domains [65,67]. For example, Bell et al. (2020) found that higher residential densities, presence of railway stations, more playgroups and kindergartens/pre-primary schools, and less backyard space were associated with decreased odds in physical development vulnerability [67]. Christian and colleagues (2017) found local communities with fewer main roads showed decreases in social and emotional competence development vulnerabilities [65].

The distribution and quality of neighbourhood-built environment features, encapsulated as ‘liveability,’ tend to differ by neighbourhood disadvantage too; less disadvantaged areas typically have a higher number and better-quality services and destinations compared with more disadvantaged areas [61]. The AEDC-BE dataset can generate evidence for built environment features that not only supports ECD, but also identifies which neighbourhood-built environment features can reduce systematic ECD inequities, and which neighbourhoods need it most. In Table 3, descriptive statistics showed that the most disadvantaged areas have smaller proportions of children with *no* local destination and service access. Though we cannot tell from our dataset whether this ameliorates the social gradient, future research could address this.

Second, the AEDC-BE provides the breadth of national coverage and ability to explore associations across approximately 80% of the estimated five-year old population in Australia. National coverage ensures the study is representative of Australia’s urban-dwelling children, and provides ample statistical power to explore modelling that accounts for real-world complexities, e.g., variations in relationships by city, state/territory, remoteness. Cities vary in their urban structure and design; population demographics and size; economic, social, and environmental conditions and issues; and policies. An Australian liveability report showed that states and territories have different urban design and planning policies for key liveability domains (e.g., walkability, public transport, public open space), and there is little evidence of cities achieving these policy targets [68]. Examining city differences and similarities in built environment and ECD relationships is not only a unique contribution to the international literature (many studies are constrained to just one or two cities), but allows context-specific information for policy-makers and practitioners about what built environment features support optimal ECD for different areas.

To illustrate one example, currently we do not know the extent to which hard (e.g., public transport) and soft (e.g., social services) ‘liveability’ infrastructure and amenity provision are being delivered in Australian cities’ outer neighbourhoods (i.e., growth corridors) compared with more established inner neighbourhoods, and whether any discrepancies in amenities play a role in ECD inequities. Much of Australia’s population growth is occurring in urban growth corridors, commonly defined as greenfield sites on undeveloped land on the outskirts of cities [69]. Many young families are moving to outer suburbs because of relative housing affordability [70]. Yet, urban fringe developments typically have less access to essential services such as schools, childcare, and health services [71]. Qualitative research with young families and engagement with policy-makers suggest that investments in infrastructure and amenity provision in urban growth corridors may not be keeping pace with population settlement [70]. The consequence is that those living on the urban fringe often spend more time travelling in cars away from the neighbourhood, and less time exploring and interacting with people locally; this can isolate children and families, and potentially negatively impact ECD [9]. Building neighbourhood design infrastructure appropriate for young children is important from the outset. Given most of the urban design and planning policies are not based on academic evidence, generating indicators as tools for policy, practice, and community use can better support ‘what’ built environment features are essential for ECD, ‘how’ and ‘how much’ they relate to ECD outcomes, and ‘where’ they are delivered. As such, indicators are intended to help monitor and guide short-, medium-, and longer-term urban planning policies and interventions that support good ECD and reduce inequitable conditions. Third, the AEDC-BE dataset presents opportunities to explore built environment and ECD relationships at small geographic levels. Creating built environment measures around each child’s residential address (individual) rather than relying on area-level ‘averages’ aggregated up to pre-defined administrative spatial units (e.g., cities, towns, suburbs, census tracts, health districts, and school areas) limits the impact of the modifiable areal unit problem and ecological fallacy [72]. Using area-level averages aggregated up to administrative spatial units can result in non-associations or inaccuracy in results (i.e., under- or over- estimation of neighbourhood-built environment effects on health and behaviour outcomes) [73]. A finer-grained approach allows ‘pockets’ of inequitable distribution of physical access to services or destinations within neighbourhoods to be mapped and identified. As such, identifying smaller areas of inequitable distribution better enables infrastructure planning for areas which may need it most. For example, if a child lives in a disadvantaged ‘pocket’ or area of the local community (with say, few services), this may impact their development and health. Examining differences between and within cities, suburbs, and neighbourhoods can potentially identify policy levers specific to different contexts. Such information can be applied to develop more precise policies to interrupt pathways of disadvantage [74].

### 4.2. Challenges and Limitations

Despite the potential of the AEDC-BE, there are limitations. In the built environment and health literature, there is an increasing emphasis placed on using context- and behaviour-specific measures. The AEDC was not purpose-designed to explore associations between neighbourhood-built environments and ECD. Instead, it was designed to be used as a population measure of early childhood development by collecting information on children’s developmental behaviours in their first year of school. Socioecological child development frameworks highlight multiple environments of influence [21]. Variables required to examine a multi-level ecological framework with ECD were not included in the AEDC (e.g., parent and family characteristics, travel to children’s activities, other community and perceived neighbourhood factors). Further linking to other data sources that collect information from multiple informants and sources may be required, e.g., children and parents, other neighbourhood attributes.

The AEDC-BE uses observational, cross-sectional data (rather than longitudinal data or natural experiments) to test associations between neighbourhood-built environments and ECD. Causal inferences (assigning cause-and-effect to observed results) based on these types of data should not be implied. Future use of longitudinal data (data on the same people that are collected over multiple time points) in built environment and ECD research would strengthen causal inference.

Whereas this study describes objective methods for identifying and quantifying built environment features using spatial data and GIS software, others have emphasised the value of qualitative data for exploring neighbourhood environments in which young children live [20,52]. Participants’ perceptions provide important insights and awareness of neighbourhood characteristics, irrespective of their objective attributes [75]. Using a mix of both subjective and objective measures of the environment is optimal, because behaviour is likely influenced by both the actual environment, as well as how it is perceived [76]. Measuring both provides insights into how to design community-level interventions, i.e., whether we need to change the built environment, target people’s perceptions, or a combination of both.

In addition, although the AEDC-BE dataset has comprehensive ‘national’ coverage of Australia′s 21 largest cities, it does not include all areas, such as smaller regional cities or remote towns. Future research should seek to explore regional- and remote-specific built environment measures. There is also further opportunity to link other relevant environment data shown to influence early childhood outcomes, for example, air pollution data [77,78].

## 5. Conclusions

This paper reflects the data linkage method used to assemble a world-first dataset of ‘individual’ spatial measures (around children’s home addresses) with national coverage population health data in Australia. It highlights the opportunities and challenges of the dataset to investigate associations between neighbourhood-built environment features and ECD at fine-grained (and aggregated) geographic levels and at scale for the first time. Such research is a step toward providing evidence-based indicators as tools for identifying and monitoring ECD-supportive neighbourhoods and informing evidence-based place-based initiatives for ECD at scale. Policy and practice implications include informing the design of community-level ECD interventions and place-based initiatives.

## Figures and Tables

**Table 1:** Examples of built environment measures available in the AEDC-BE.

Feature	Built Environment Measures
Destinations and services	Count of destination type, any distance up to 3200 m. Closest distance (m) to destination from parcel/lot address to child care centre, library, general practitioner, preschool, primary school, maternal and child health centre, food outlet, sporting facility, swimming pool, community centre. Quality of early childhood education and care (ECEC) services: e.g., derived from the Australian Children’s Education & Care Quality Authority (ACECQA) data.
Public open space (POS)	POS types: park, public school ground, natural areas, beach. Count of each POS type, any distance up to 3200 m. Closest distance (m) to each POS type; + park attributes: playground, sport ground, water feature, amenities (toilet, picnic area); + POS *size* (any, ≥0.5 ha; ≥1 ha; ≥1.5 ha; ≥2 ha; ≥5 ha).
Walkability/connectivity	Walkability and street connectivity, 1600 m. Street connectivity per square kilometre for 1600 m street network distance Walkability refers to daily living destination access score for 1600 m street network distance. The daily living score refers to a broader set of neighbourhood destinations that people might regularly visit. The presence or absence is 0 or 1 for the following 11 destinations within 1600 m, and summed to provide a score between 0–11: (1) convenience store, supermarket; (2) public transport stop; (3) speciality food (e.g., fruit, veggie, meat, fish); (4) post-office; (5) bank; (6) pharmacy; (7) general practitioner/medical centre; (8) dentist; (9) community centre/hall; (10) child care facility; (11) library. Walkability traditionally combines a land use mix, street connectivity, and residential density. LUM is difficult to calculate at a national level due to a lack of data. There have been issues with the LUM entropy measure; hence, the ‘daily living score’ is used as a replacement; this has been validated in another study [54].
Public transport	Public transport stop = ferry, tram, train, bus Count of public transport stops, any distance up to 3200 m. Closest distance (m) to public transport stop. Frequency of public transport: % of residential dwellings within 400 m of a public transport stop with a scheduled service at least every 30 min between 7 am and 7 pm on a normal weekday.
Housing	Housing stress: Average percentage of SA1 households with income in the bottom 40% of the income distribution spending more than 30% of household income on housing costs. Renters: % of residential dwellings renting as a proportion of total in area. Type: % of separate house, semi-detached, units/apartments.
Traffic	Length of different road volume types (m) to busy roads within the area.

SA1: Statistical area level 1 [50]. Refer to the Australian Urban Observatory for more information [53]: https://auo.org.au/portal/metadata (accessed on 8 March 2022).

**Table 2:** Sample demographics of the AEDC-BE by child development outcomes.

	Not Developmentally Vulnerable		Developmentally Vulnerable on at Least One Domain (DV1)		Missing Development Outcome		Overall Sample	
	n	%	n	%	n	%	n	%
Age group ^a^								
Under 5 years	651	74.9	173	19.9	45	5.2	869	100
5 years	128,733	74.5	36,866	21.3	7139	4.1	172,738	100
6+ years	46,752	75.3	10,377	16.7	4919	7.9	62,048	100
Gender								
Female	94,639	82.39	16,410	14.29	3816	3.32	114,865	100
Male	81,467	67.47	31,006	25.67	8287	6.86	120,790	100
Maternal education ^b^								
Year 9 or less	3448	53.9	2540	39.7	404	6.3	6392	100
Year 10	7584	62.6	3737	30.9	792	6.5	12,113	100
Year 11	4556	65.9	1959	28.4	395	5.7	6910	100
Year 12 or more	147,598	77.3	34,382	18.0	8932	4.7	190,912	100
Missing	12,950	67.0	4798	24.8	1580	8.2	19,328	100
SEIFA-IRSD of SA1								
Q1 Most disadvantaged	24,128	64.2	10,945	29.1	2503	6.7	37,576	100
Quintile 2	27,876	70.4	9402	23.8	2301	5.8	39,579	100
Quintile 3	33,674	74.9	8965	19.9	2300	5.1	44,939	100
Quintile 4	40,964	78.1	9006	17.2	2463	4.7	52,433	100
Q5 Least disadvantaged	48,834	81.1	8907	14.8	2498	4.1	60,239	100
Missing/not applicable	660	74.2	191	21.5	38	4.3	889	100
Aboriginal and Torres Strait Islander								
No	171,411	75.3	44,701	19.6	11,432	5	227,544	100
Yes	4725	58.3	2715	33.5	671	8.3	8111	100
Language background other than English								
No	134,466	76.2	32,410	18.4	9517	5.4	176,393	100
Yes	41,670	70.3	15,006	25.3	2586	4.4	59,262	100
Child has special needs								
No	176,136	78.4	47,416	21.1	1238	0.6	224,790	100
Yes	-	-	-	-	10,865	100	10,865	100
Local community area remoteness category ^c^								
Major Cities	157,000	74.9	41,782	19.9	10,766	5.1	209,548	100
Regional (inner or outer)	19,116	73.3	5631	21.6	1336	5.1	26,083	100
State/Territory								
Australian Capital Territory	3989	73.8	1157	21.4	258	4.8	5404	100
New South Wales	55,823	76.1	13,783	18.8	3748	5.1	73,354	100
Northern Territory	1340	70.3	458	24.0	107	5.6	1905	100
Queensland	35,612	71.2	11,991	24.0	2411	4.8	50,014	100
South Australia	10,968	72.4	3270	21.6	919	6.1	15,157	100
Tasmania	2955	76.1	769	19.8	157	4.0	3881	100
Victoria	45,623	76.0	10,995	18.3	3443	5.7	60,061	100
Western Australia	19,826	76.6	4993	19.3	1060	4.1	25,879	100
**Total**	176,136	74.7	47,416	20.1	12,103	5.1	235,655	100

AEDC-BE: Australian Early Development Census-Built Environment dataset using 2015 AEDC data. ^a^ Age groups were derived from 15 age categories. The ‘5 years’ age group includes some children slightly less than 5 years old (ages 4 years 10 months and older), and the ‘6 years and older’ group includes children aged 5 years 10 months and older. ^b^ Based off the variable ‘parent 1 schooling’ where parent 1 is the main contact for the child. Previous Australian data show that this is almost always the child’s mother. ^c^ Remote or very remote residences are still possible because of local government area zoning changes (not reported due to data suppression guidelines).

**Table 3:** Proportion of children in Australia’s major and regional cities with *no* (zero) local destinations/services by neighbourhood disadvantage.

	Destinations 1600 m ^1^ from Child’s Home Address								Destinations 3200 m ^1^ from Child′s Home Address											
Neighbourhood Disadvantage	Public Transport n (%)		Public Open Space (POS) n (%)						Early Childhood Education and Care Services n (%)				Family-Friendly Destinations n (%)						Food Outlets n (%)	^#^ Total
	No Public Transport Stops	No Frequent Public Transport Stops	No POS	No POS ≤ 0.4 Ha	No POS > 0.4 to ≤ 1 Ha	Count of POS ≤ 0.4 Ha	No POS > 1.5 Ha	No Playgrounds	No Childcare Centres Meeting National Standards	No Childcare Centres Exceeding National Standards	No Preschool Services Meeting National Standards	No Preschool Services Exceeding National Standards	No Sport Facilities	No Public Swimming Pools	No Public Libraries	No Community Centres	No Activity Centres	No Family-Friendly Destinations	No Healthier Food Outlets	
**Major City (n = 182,913), n (%) Without Access**																				
**Q1**	14 (0.1)	4560 (16.3)	55 (0.2)	2392 (8.5)	2731 (9.8)	66 (0.2)	154 (0.6)	2871 (10.3)	47 (0.2)	659 (2.5)	1104 (4.2)	3763 (14.4)	56 (0.2)	8255 (29.5)	5555 (19.8)	10,105 (36.1)	1945 (6.9)	44 (0.2)	73 (0.3)	28,023 (100.0)
**Q2**	203 (0.7)	7461 (25.1)	153 (0.5)	2990 (10.1)	3541 (11.9)	173 (0.6)	330 (1.1)	2902 (9.8)	230 (0.9)	1320 (5.0)	2233 (8.5)	4514 (17.1)	141 (0.5)	10,840 (36.5)	9179 (30.9)	15,258 (51.3)	4337 (14.6)	121 (0.4)	284 (1.0)	29,741 (100.0)
**Q3**	562 (1.6)	9726 (28.3)	361 (1.1)	3593 (10.5)	4134 (12.0)	416 (1.2)	678 (2.0)	3334 (9.7)	473 (1.6)	1859 (6.1)	3078 (10.1)	5550 (18.2)	584 (1.7)	12,465 (36.2)	12,285 (35.7)	17,734 (51.6)	5419 (15.8)	456 (1.3)	325 (1.0)	34,391 (100.0)
**Q4**	1049 (2.5)	11,854 (28.7)	536 (1.3)	4418 (10.7)	5153 (12.5)	589 (1.4)	937 (2.3)	4620 (11.2)	789 (2.2)	2208 (6.2)	4155 (11.6)	6724 (18.8)	1097 (2.7)	15,480 (37.5)	15,697 (38.0)	20,904 (50.6)	7790 (18.8)	813 (2.0)	481 (1.2)	41,339 (100.0)
**Q5**	1291 (2.6)	14,961 (30.3)	414 (0.8)	4839 (9.8)	6546 (13.3)	480 (1.0)	764 (1.6)	5117 (10.4)	880 (2.1)	2058 (4.9)	3920 (9.3)	6435 (15.3)	811 (1.6)	16,570 (33.5)	17,771 (36.0)	25,461 (51.5)	8954 (18.1)	599 (1.2)	536 (1.1)	49,419 (100.0)
^ Total	3119 (1.7)	48,562 (26.6)	1519 (0.8)	18,232 (10.0)	22,105 (12.1)	1724 (0.9)	2863 (1.6)	18,844 (10.3)	2419 (1.5)	8104 (5.0)	14,490 (9.0)	26,986 (16.7)	2689 (1.5)	63,610 (34.8)	60,487 (33.1)	89,462 (48.9)	28,445 (15.6)	2033 (1.1)	1699 (0.9)	182,913 (100.0)
**Regional City (n = 22,117), n (%) Without Access**																				
**Q1**	285 (8.0)	2266 (63.7)	151 (4.3)	981 (27.6)	1042 (29.3)	158 (4.4)	236 (6.6)	1493 (42.0)	182 (6.6)	409 (14.8)	271 (9.8)	647 (23.4)	177 (5.0)	1322 (37.2)	1247 (35.1)	1924 (54.1)	953 (26.8)	120 (3.4)	25 (0.7)	3556 (100.0)
**Q2**	641 (14.6)	3220 (73.4)	376 (8.6)	1613 (36.8)	1416 (32.3)	398 (9.1)	548 (12.5)	1823 (41.6)	520 (13.7)	841 (22.2)	721 (19.0)	1195 (31.5)	497 (11.3)	2138 (48.7)	2332 (53.2)	2953 (67.3)	1631 (37.2)	458 (10.4)	63 (1.4)	4387 (100.0)
**Q3**	1203 (23.9)	4050 (80.5)	827 (16.4)	2272 (45.2)	2171 (43.2)	875 (17.4)	1075 (21.4)	2228 (44.3)	923 (21.6)	1646 (38.6)	1384 (32.4)	2117 (49.6)	945 (18.8)	3050 (60.6)	3381 (67.2)	3871 (76.9)	2609 (51.9)	875 (17.4)	100 (2.0)	5031 (100.0)
**Q4**	1168 (23.0)	4206 (83.0)	882 (17.4)	2242 (44.2)	2475 (48.8)	961 (19.0)	1110 (21.9)	2441 (48.2)	1191 (27.6)	1907 (44.2)	1720 (39.8)	2419 (56.0)	1195 (23.6)	3343 (66.0)	3784 (74.7)	4163 (82.1)	3045 (60.1)	1128 (22.3)	189 (3.7)	5069 (100.0)
**Q5**	962 (23.6)	3565 (87.5)	578 (14.2)	1857 (45.6)	1998 (49.0)	665 (16.3)	818 (20.1)	2155 (52.9)	1016 (27.5)	1541 (41.6)	1416 (38.3)	1942 (52.5)	795 (19.5)	2549 (62.6)	3099 (76.1)	3410 (83.7)	2491 (61.1)	760 (18.7)	171 (4.2)	4074 (100.0)
^ Total	4259 (19.3)	17,307 (78.3)	2814 (12.7)	8965 (40.5)	9102 (41.2)	3057 (13.8)	3787 (17.1)	10,140 (45.9)	3832 (20.3)	6344 (33.7)	5512 (29.2)	8320 (44.1)	3609 (16.3)	12,402 (56.1)	13,843 (62.6)	16,321 (73.8)	10,729 (48.5)	3341 (15.1)	548 (2.5)	22,117 (100.0)

Key: Q: quintile, Q1 = most disadvantaged, Q5 = least disadvantaged. ^1^ Walkable street network distance. ^ Overall number of children with no access to destination type. ^#^ Overall number of children in neighbourhood disadvantage quintile.

## Data Availability

The linked dataset may be available for research purposes pending appropriate data use approvals from the ethics committee, data custodians, and research team. For more information, please contact: hannah.badland@rmit.edu.au.

## Supplementary Materials

The following supporting information can be downloaded at: https://www.mdpi.com/article/10.3390/ijerph19095549/s1, Table S1: Built environment data sources.
