# Supplementary material for: Data to Decisions: Methods to Create Neighbourhood Built Environment Indicators Relevant for Early Childhood Development
Source: Int J Environ Res Public Health. 2022 May 3;19(9):5549. doi: 10.3390/ijerph19095549 (PMC9102076; doi:10.3390/ijerph19095549)
Supplement: Supplementary file 1 [file ijerph-19-05549-s001.zip › ijerph-1651942-supplementary.pdf]

Supplementary Material Table S1. Built environment data sources

Custodian	Year	Data source description	Purpose
**Department of Education, Skills and Employment**	2015	Australian Early Development Census	Child development data, child demographics including data relating to geography derived using publicly available ABS sources (SEIFA-IRSD of child's SA1, ASGS remoteness category)
**ABS**	2016	Census of population and housing, customised data report	Housing affordability
	2016	ASGS Volume 1 geopackage	Mesh Block, SA1, SA2, SA3, SA4, GCCSA boundaries
	2016	Mesh Block dwelling and person counts	Dwellings
	2016	Mesh Block - Suburb linkage csv	Suburb
	2016	Mesh Block - LGA linkage csv	LGA
	2016	SA1 urban centres and localities linkage	Section of State
	2016	ASGS Volume 3 geopackage	Suburb and LGA geometries
	2016	ASGS Volume 4 geopackage	Significant Urban Area geometries
	2016	SEIFA IRSD 2011 (SA1)	IRSD related statistics
**OpenStreetMap**		Retrieved 1 October 2018	
	2018	Pedestrian network, generated using OSMnx, using Overpass API using hybrid walk-cycle network	Accessibility analysis
	2018	Intersections, generated using OSMnx with processed pedestrian network	Modelling street connectivity
	2018	Destinations (see definitions, elsewhere)	Accessibility analysis
	2018	Open Space (see definitions, elsewhere)	Accessibility analysis
**ACECQA**	2019	Australian Children's Education & Care Quality Authority child care centres (geocoded)	Accessibility analysis
**ACARA**	2019	Australian Curriculum, Assessment and Reporting Authority (ACARA), Primary and secondary schools, by sector (geocoded)	Accessibility analysis

**NHSD**	2017	National Health Services Directory (via AURIN Portal)	Accessibility analysis
**Pitney Bowes**	2014	Convenience stores	Accessibility analysis
**Pitney Bowes**	2014	Newsagencies	Accessibility analysis
**Pitney Bowes**	2014	Petrol stations	Accessibility analysis
**HLC**	2017	Additional geocoded datasets curated by Health Liveable Cities group from multiple sources (supermarket major chains, 2017; Australian Libraries, 2016-18)	Accessibility analysis
**PSMA**	2017	Transport and Topography	Traffic exposure
**State Transport agencies**	2018	GTFS feed data covering the period 8 October to 5 December for 2018	Accessibility and transport analysis

Key: ABS: Australian Bureau of Statistics. ACECQA: Australian Children's Education & Care Quality Authority. ASGS: Australian Standard Geography Structure. GCCSA: Greater Capital City Statistical Area. GTFS: General Transit Feed Specification. HLC: Healthy Liveable Cities Group (RMIT). LGA; Local Government Authority. NHSD: National Health Services Directory. SA1: Statistical Area Level 1; SA2: Statistical Area Level 2; Statistical Area Level 3; SA4: Statistical Area Level 4. SEIFA-IRSD: Socio-Economic Index For Areas – Index of Relative Socio-Economic Disadvantage.
